# Technological Perception with Rural and Urban Differentiation and Its Influence on the Quality of Life of Older People with Age-Related Macular Degeneration

**DOI:** 10.3390/ejihpe14050097

**Published:** 2024-05-20

**Authors:** Angel Parra-Sanchez, Vanessa Zorrilla-Muñoz, Gema Martinez-Navarrete, Eduardo Fernandez

**Affiliations:** 1 Neuroprosthesis and Visual Rehabilitation Laboratory, Bioengineering Institute, University Miguel Hernández of Elche, 03202 Elche, Spain; a.parra@umh.es (A.P.-S.); e.fernandez@umh.es (E.F.); 2 Bioengineering Institute, University Miguel Hernández of Elche, 03202 Elche, Spain; 3 Institute on Gender Studies, University Carlos III of Madrid, Getafe, 28903 Madrid, Spain; 4 Biomedical Research Network Center (CIBER-BBN), 28029 Madrid, Spain

**Keywords:** AMD, rural, sex, older people, self-perceived health, new technologies, quality of life

## Abstract

The past decade has seen a global increase in population age, especially in developed countries, where aging involves visual diseases such as age-related macular degeneration (AMD), which severely affect quality of life (QoL) and mental health, as well as increase isolation and care costs. This study investigated how persons with AMD perceive the impact of technology use on their QoL, focusing on potential disparities between urban and rural contexts in Spain. Using a cross-sectional observational design, data from the 2020 National Statistics Institute’s Disability, Personal Autonomy, and Dependency Situations Survey were analyzed, focusing on QoL aspects based on the WHO items of the WHOQOL-100 scale. The results revealed a generally positive perception of technology among participants, with urban residents perceiving technology’s positive impact more favorably. Sex discrepancies in technology perception were also observed, as women exhibited a more positive outlook on technology’s influence on QoL. The analysis of QoL aspects, such as ‘Visibility’, ‘Learning’, ‘Mobility’, and ‘Domestic life’, highlighted distinct challenges faced by rural and urban populations, underscoring the importance of context-specific approaches in technology interventions. However, these perceptions were intertwined with comorbidities, which can exacerbate AMD-related issues. Furthermore, this study explored the role of technology in enhancing QoL among older adults with AMD, examining how it influences daily activities and independence, particularly in the context of AMD management. This study concluded that developing more-inclusive policies tailored to the specific needs of persons with AMD, with special attention to environmental and sex differences, is imperative to enhance the positive impact of technology on their QoL.

## 1. Introduction

**Figure 1:**
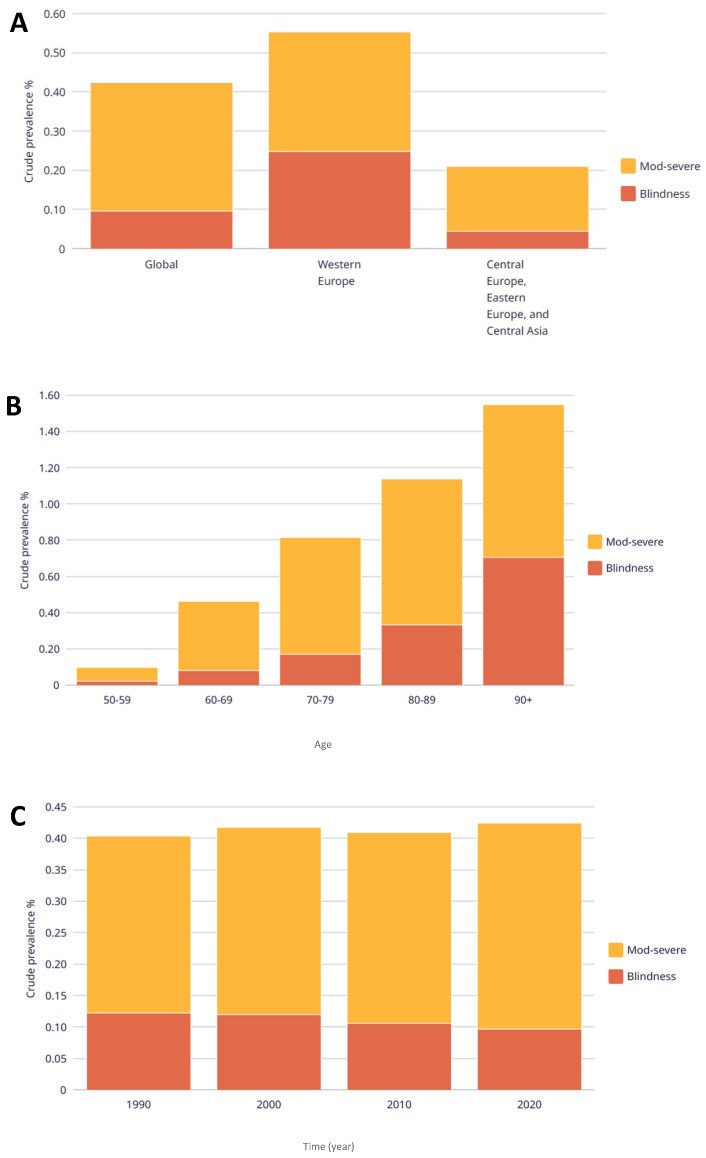
Crude prevalence of vision loss due to age-related macular degeneration by location (**A**), age (**B**), and time period (**C**). Adults ≥ 50 years old, men and women. Source: Data from VLEG/GBD 2020 model, accessed via the IAPB Vision Atlas (https://www.iapb.org/learn/vision-atlas/magnitude-and-projections/gbd-super-regions/, accessed on 2 February 2024).

In the last decade, there has been an increase in the global population’s age, especially in developed countries, such as the European community, where more than 17% of the total population is over 65 years old [1]. This increase in age has led to the emergence of diseases related to visual impartments and blindness (Figure 1). Visual impairment and blindness in older adults significantly impact quality of life (QoL) and mental health and may lead to anxiety and depression [2,3,4,5]. Moreover, persons with visual disabilities and/or blindness may experience isolation, stigma and self-stigma, discrimination, and other social conditions affecting well-being [6,7,8]. For older adults, visual impairment can reduce physical activity [9], increase the risk of hip fracture [10], and contribute to social isolation [11]. Additionally, older people with low vision are more likely to be admitted to nursing homes, increasing the cost of care [10,12] and the burden related to informal support/care by relatives [13]. This represents a significant global burden, with an estimated annual loss of worldwide productivity amounting to approximately USD 411.3 million. This figure far exceeds the estimated cost for addressing the needs of visual impairment [14,15].

Visual impairment and blindness in aging is associated with main diseases such as glaucoma, diabetic retinopathy, and age-related macular degeneration (AMD). AMD is the most common cause of blindness in developed countries, accounting for 8.7% of all blindness worldwide [16,17,18,19], reaching around 196 million cases in 2020, a figure expected to increase to 288 million by 2040 [20]. AMD is a chronic and progressive degenerative disorder that primarily affects the central part of the retina known as the macula. It is characterized by the loss of central vision due to abnormalities in the photoreceptor/retinal pigment epithelium/Bruch’s membrane/choroid complex, often leading to geographic atrophy and/or neovascularization. AMD can be classified into dry and wet forms, with the latter being responsible for the majority of the severe vision loss [21]. The prevalence of AMD-related vision loss is higher in western Europe than in other regions and countries worldwide, with a greater prevalence among older people. Over the past decade, the prevalence of AMD has slightly increased. In Spain, data from the National Institute of Statistics (INE) disabilities database [22] indicate that AMD disproportionately affects women as they age, with a prevalence of 6.09 per 1000 inhabitants among those aged 65 to 69 years and 27.22 per 1000 inhabitants aged 80 and older. In comparison, the prevalence among men in the corresponding age group is 3.09 per 1000 and 17.35 per 1000 inhabitants, respectively. Therefore, AMD is a disease that is more closely associated with women, placing them at a higher risk, thus suggesting the need for a sex-based analysis. According to sex in AMD, some authors such as Lin et al. suggested sex disparities and inequality burdens associated with such diseases [23].

The risk factors for AMD can be broadly classified into two main groups: (1) environmental factors and (2) personal factors. Environmental factors include (a) smoking, (b) exposure to sunlight, and (c) nutritional factors such as a lack of micronutrients, fish-poor diets, and alcohol consumption. Environmental factors encompass not only external physical elements, such as pollution and climate, but also aspects of the social and cultural environment that significantly affect individual decisions and behaviors. For example, the practice of smoking is influenced by the social and cultural environment, such as advertising and social norms. Additionally, smoking introduces toxins that are known risks to ocular health [24]. On the other hand, personal factors can be further subdivided into (a) sociodemographic factors (age, sex, race/ethnicity, heredity, and socioeconomic level), (b) ocular factors (mainly iris color, optical density of the macular pigment, cataracts and their surgery, refractive error, and cup/disc ratio), and (c) systemic factors (suffering cardiovascular disease, reproductive system disorders, hormonal problem, elastotic degeneration of the skin, and antioxidant enzyme scarcity) [21]. Focusing on the risk factors evaluated in this study, Święch et al. found that the place of residence (urban or rural) and sex can influence the perception of the exudative form of AMD [25]. This perception can impact the QoL and well-being of older people, especially those over the age of 50 years. Additionally, AMD has been found to be associated with rural regions [26,27], especially when comparing urban and rural populations for an intervention. These personal factors include demographics, health literacy, cultural beliefs and attitudes, psychological factors, lifestyle and behavior, economic stability and technological proficiency. Overall, while sex-related factors may contribute to AMD risk, the interplay between genetic, lifestyle, and environmental factors likely plays a more significant role in determining individual risk profiles [28].

AMD, an irreversible condition, is currently addressed with innovative technologies with therapies like ultra-wide-field fundus ophthalmoscopy-assisted deep learning [29] and microarray technology [30], alongside established treatments such as photodynamic and anti-VEGF therapies [31], which aim to mitigate vision loss. Despite these advancements, not all forms of vision loss are preventable, and disparities in access to treatments like anti-VEGF injections persist [32,33]. Consequently, there is an increasing focus on the socio-health impacts of technology [34] and the need for research into the usability and acceptability of these technologies, particularly in patients with AMD [35,36], to enhance QoL and safety. Given these factors, it is likely that disruptive technologies and new therapies and drugs will be developed in the future for the treatment of AMD with the potential to improve QoL. In this context, authors from the last century, such as Williams (1998), found that persons affected by AMD experience emotional distress and a reduced QoL in their daily activities. Moreover, these persons usually report lower self-rated general health due to their low visual acuity [37]. Mitchell and Bradley (2006) highlighted the significant decrease in QoL experienced by persons with AMD when there is a delay in diagnosis and a lack of ongoing medical support. They concluded that investing in rehabilitation, low-vision aids, training, and ongoing support could have a significant impact on QoL [38]. More recently, COVID-19 has served as an example of the lack of support and delays in medical treatments for conditions like AMD. Sanabria et al. (2023) demonstrated a worsening perception of QoL in those with AMD [39]. According to Van Hu et al. (2023), comorbidities are factors influencing QoL, with the presence of ≥3 chronic diseases in addition to AMD being associated with lower evaluations of the mental health component. This can lead to depressive symptoms and a deterioration in basic activities of daily living [40]. A series of studies have explored the impact of AMD on QoL using different methodologies, consistently revealing significant declines in QoL among affected patients. A study employing the National Eye Institute Visual Function Questionnaire-25 (NEI-VFQ-25) found that patients with AMD reported lower scores across all domains of the questionnaire, highlighting a substantial negative impact on their QoL [41]. Similarly, research utilizing the International Index of Quality of Life (IQoL) observed that patients with AMD scored lower in both the physical and emotional domains, further emphasizing the profound effects on the overall quality of life [42]. Additionally, an analysis focusing on the correlation between visual acuity and QoL in Patients with AMD revealed that those with lower visual acuity experienced significantly reduced QoL, underscoring the direct relationship between the severity of vision loss and QoL degradation in these individuals [43].

In summary, previous studies have examined the concept of QoL from multiple and interdisciplinary perspectives, considering various factors that impact the daily activities and independence of patients with AMD. However, it remains unclear whether persons affected by AMD perceive the positive impact on their QoL when using technology as a means of support and assistance. Therefore, it is essential to gain a comprehensive understanding of the overall perception of these people in order to develop technologies that can facilitate the management and support of their condition, ultimately improving their QoL.

Taking this perspective into account, the assessment of QoL among older people affected by AMD becomes significant in relation to the role of technology and sociodemographic variables. Based on these previous considerations, the objective of this study was to determine the perspective and influence of technology on QoL determinants among adults and older persons (≥50 years old) with AMD, residing in both rural and urban areas of Spain, using data obtained from the Survey of Disability, Personal Autonomy and Dependency Situations, collected by the National Statistics Institute (INE).

## 2. Materials and Methods

This research was based on examining technological perception and its influence on the quality of life among people 50 years and older affected by AMD.

### 2.1. Data Collection

This study was based on data from the “Survey of Disability, Personal Autonomy and Dependency Situations” (AGE) [22] developed by the National Institute of Statistics (INE). All ethical considerations and data protection measures were handled and documented by the INE. Also, this study received approval from the Ethics Committee of the Miguel Hernández University of Elche (code UT.IB.GCMN.240202). AGE encompasses a comprehensive macro-survey that specifically targets persons residing in private households within Spain. The questionnaire was administered during the year 2020 and has already undergone rigorous validation processes conducted by the INE. The data collected included as criteria for disability or limitation needed to be current and have a duration at least one year. The sample size consisted of 68,000 Spanish households. Furthermore, in order to achieve the survey’s objectives and ensure reliable estimations at the national and regional levels, the INE determined an initial sample size of 110,130 households distributed across 3671 census sections. This decision to consider a sample of 110,130 households was based on the sample obtained from a previous disability survey conducted in 2008. Each section had thirty households selected as titular units [22]. Data collection involved a two-stage stratified approach, employing a multidata collection method that included computer-assisted web interviewing (CAWI) and computer-assisted personal interviewing (CATI) collection, and a paper questionnaire sent by regular mail. When persons with disabilities were identified, their information was collected through a CAPI interview using an individual questionnaire, which means that each respondent answered questions in a personalized interview. However, due to the outbreak of the COVID-19 pandemic during the field work, the data had to collected through CAPI and CATI in a second phase, resulting in a reduction in the initial sample (from 110,130 to 68,000 Spanish households). The survey aimed to capture the experiences of approximately 4.38 million people (equivalent to 94.9 per 1000 inhabitants) who had reported having some form of disability. The figure of 4.38 million individuals reporting some form of disability is an estimate applied to the sample data, adjusted for the probability of selection, nonresponse, and poststratification factors to ensure that the estimates were representative of the national population. This was calculated with a precision of 4.3 decimals, as indicated by the INE in its methodology (https://www.ine.es/metodologia/t15/meto_edad_2020.pdf, accessed on 2 February 2024, https://www.ine.es/daco/daco42/discapa/faltar_2020.pdf, accessed on 2 February 2024) to ensure the accuracy of these adjustments. The detailed level of precision helped to accurately scale the sample data to reflect the entire population, taking into account the complex survey design.

### 2.2. Sampling

The total sample size corresponded to 2405 individuals (see Table 1). The sample size was justified as follows: the prevalence of AMD in the Spanish population was 3.4% in 2022 [44], while data collected by the INE in 2020 included a total of 21,021,128 individuals aged 50 years or older [22]. Considering 21,021,128 individuals with a prevalence rate of 3.4%, there were 714,718 individuals affected by AMD and 20,306,410 individuals at risk of developing AMD in 2020. Based on this, the sample of 367 individuals with AMD was obtained with a confidence level (1–α) of 95% (with noncritical consideration), a precision (d) of 3%, a probability (*p*) of 0.3, and an expected loss proportion (R) of 12% for 714,718 persons with AMD. The calculated sample size for 20,306,410 persons without AMD was 2038 individuals for 1-α = 95%, d = 3%, *p* = 0.5, and R = 47.64%.

**Figure 2:**
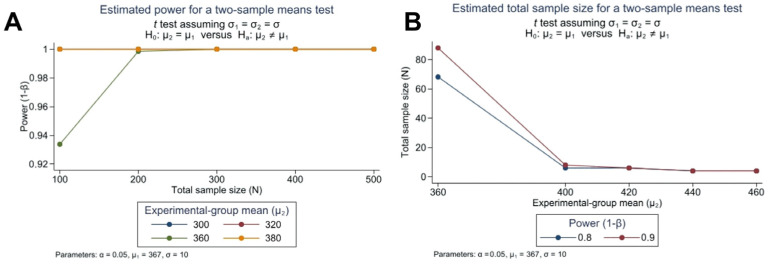
Power range (**A**) and total sample size representation (**B**) for the AMD sampling.

For the AMD sampling, we assumed 367 persons with a standard deviation of 10. First, we calculated the power obtained for sample size increases by 20, 40, 60, and 80 points when population increases 100, 200, 300, 400, and 500. The power range obtained was 0.92–1 (Figure 2A). Second, the power calculation was performed to detect the increased population with a standard deviation of 10 at power levels of 0.8 and 0.9 (Figure 2B).

Data were extracted regarding persons aged 50 years or more and persons aged more than 50 years at risk of developing AMD. For this reason, the sample included diagnosed and undiagnosed persons 50 years and older living in rural and urban areas, diagnosed with AMD by a health professional, with their relationship to other visual pathologies and other diagnoses related or unrelated to vision pathologies in rural and urban areas (see Table 2). The total sample included 2405 persons, where a total of 53.13% of persons with AMD had been diagnosed with cataracts. Moreover, there were more comorbidities for persons with AMD, such as osteoarthritis (60.22% of persons diagnosed with AMD) and arthritis (33.79%). Regarding rural persons affected by AMD, cataracts cover a total of 51.38%, osteoarthritis 55.96%, and arthritis the 33.11%. In comparison, the percentage of these diseases was slightly higher for persons who were living with AMD in urban areas: cataracts in 55.88%, osteoarthritis percentage in 62.02%, and arthritis in 34.50% (Table 2).

### 2.3. Method Description

The methodology employed for the questionnaire encompassed various aspects related to QoL based on the WHO items of the WHOQOL-100 scale (https://www.who.int/tools/whoqol/whoqol-100, accessed on 5 February 2024). This questionnaire primarily aims to elucidate an individual’s physical health, physiological state, independence level, and social relationships and their environment (https://www.ine.es/metodologia/t15/meto_edad_2020.pdf, accessed on 2 February 2024). Specifically, this questionnaire examines the domains of the level of independence, which consists of mobility, self-care, and domestic life, as well as social relationships, which encompass interpersonal relationships related to communication and learning.

The questionnaire variables were measured using a Likert scale, with varying levels as follows: The dependent variable on the perception of technology and its relationship with the improvement in the QoL was formulated in this way: ‘Do you think that the use of new technologies has improved aspects of your daily life?’ This question could be answered 1 to 4 points, with 1 indicating ‘Yes, it has improved a lot’, 2 indicating “Yes, it has improved something my daily life”, 3 indicating “Technology has not gotten better or worse my daily life”, and 4 indicating ‘No, daily life has worsened’.

Moreover, the questionnaire also incorporates independents variables: ‘Do you find difficulties for/to…’ for activities related to the items ‘Visibility’, ‘Communication’, ‘Learning’, ‘Mobility’, ‘Self-care’, ‘Domestic Life’, and ‘Interpersonal Relationships (see Table S1). A Likert scale, ranging from 1 to 3 points, was employed to measure responses, where 1 indicated a significant improvement, while a response of 3 suggested a maximum difficulty value.

### 2.4. Statistical Analysis

First, internal consistency was measured by Cronbach’s alpha coefficient, which was measured as 0.79. Second, nonparametric and parametric test was used: The Spearman correlation was used prior to applying parametric tests in order to verify the relationship between two variables when it could not be assumed that they followed a normal distribution. This evaluates the monotonic relationship between variables, meaning it does not assume a linear relationship and is less sensitive to outliers and deviations from normality in the data. The results allow confirming that the variables are monotonically correlated, establishing a stronger basis for applying parametric tests and making assumptions about the distribution of the data. This helps ensure the validity of results obtained through subsequent parametric tests. Spearman correlation nonparametric test showed that population data were normally distributed. That confirmed the validity of the use of parametric tests. Third, the Pearson correlation matrix and one-way analysis of variance (ANOVA) were analyzed for each independent variable and the dependent variable related to the specified items: ‘Visibility’, ‘Communication’, ‘Learning’, ‘Mobility’, ‘Self-care’, ‘Domestic Life’, and ‘Interpersonal Relationships’. All independent variables without significant Pearson and ANOVA values were excluded from the next analysis. Fourth, the significant variables (*p* < 0.05) in the Pearson and ANOVA tests were distributed across item variables. Fifth, Pearson correlation and ANOVA were applied between the dependent variable and each independent item formed. Significance levels of *p* < 0.01 and *p* < 0.05 are marked in the results. Sixth, the average covariance between items was measured with an average interitem covariance between 0.15 and 0.5. Seventh, the scale reliability coefficient was considered acceptable with a value of between 0.6 and 0.7 and greater than 0.8; Cronbach’s alpha coefficient, which assesses the internal consistency of the scale, was calculated based on these interitem covariances. This coefficient quantifies the extent to which all items of the scale measure the same underlying construct, with values falling within the specified ranges indicating acceptable to excellent reliability. Finally, missing data were not imputed in the model but were excluded from the analysis. This decision was made to avoid potential biases that could arise from imputing values that may not accurately represent the true responses of the participants.

All the independents variables were tested with the dependent variable ‘Do you think new technology use has improved aspects of your daily life?’ with 6 possibilities: Persons with AMD (n = 367), non-AMD persons (n = 2038), persons with AMD in rural areas (n = 109), non-AMD persons in rural areas (n = 746), persons with AMD in urban areas (n = 258,) and non-AMD persons in urban areas (n = 1292). The statistical analysis was conducted, employing STATA software (version MP 17.0, developed by Statacorp).

## 3. Results

The analysis of the data from 2405 participants demonstrated significant patterns in the perception of technology and its influence on the quality of life for people over 50 years old, with special attention to those affected by AMD. The participants were classified based on whether they lived in rural or urban areas and whether they had AMD or not.

**Figure 3:**
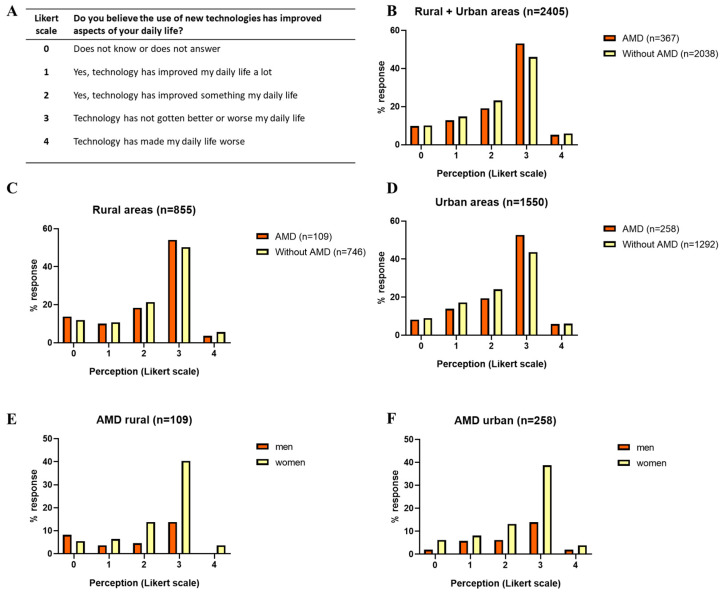
Perception of quality of life with the use of technologies in people with or without AMD in rural and urban areas in Spain. (**A**) Likert scale, (**B**) response rate in rural + urban areas, (**C**) response rate in rural areas, (**D**) response rate in urban areas, (**E**) response rate by sex in rural areas (n = 109), (**F**) response rate by sex in urban areas (n = 258).

Among the respondents, 14.51% (349 persons) of the total sample firmly believed that technology had greatly improved their daily life. This positive perception was more prevalent in the non-AMD group, where 14.82% (302 persons) expressed this view, compared to 12.81% (47 persons) of those with AMD (Figure 3A,B). The differences between rural and urban areas were also notable. In rural areas, only 10.09% (11 persons) of respondents with AMD considered that technology had significantly improved their daily life, in contrast to 13.95% (36 persons) in urban areas. Among non-AMD participants, this perception increased to 17.18% (222 persons) in urban areas, compared to 10.72% (80 persons) in rural areas (Figure 3C,D).

Moreover, 22.54% (542 persons) of the 2405 participants indicated technology had somewhat improved their daily life. This trend remained consistent among groups with and without AMD, as well as between rural and urban areas, suggesting a generally positive perception of technology. Across all scenarios, it is noteworthy that the impact of new technologies had a more pronounced positive or negative influence in urban areas than in rural ones, with a significant portion of the AMD population in urban settings reporting such effects. These results underscored the importance of technology in improving the quality of life of older adults, with a more positive perception in urban areas and among those without AMD. However, they also highlighted the necessity of addressing disparities in perception and access to technology among these groups, particularly for the purpose of improving the lives of older adults with AMD in rural areas.

Figure 3E,F illustrate the differences in technological perception between women and men, both in persons with AMD and in those without this condition. In general, women usually reported a more positive perception of the influence of technology on their quality of life compared to men. For instance, in the group of persons with AMD, there was higher percentage of women who expressed that technology had significantly improved their daily life compared to men. This trend remained constant in both urban and rural environments.

Table 3 provides data on the average interitem covariance, number of items, and scale reliability coefficients for items related to QoL, such as ‘Visibility’, ‘Communication’, ‘Learning’, ‘Mobility’, ‘Selfcare’, ‘Domestic life’, and ‘Interpersonal relationships’. The average interitem covariance for AMD items (n = 367) achieved acceptable levels, with the ‘Domestic life’ item exhibiting a coefficient of 0.3815. However, the items ‘Communication’, ‘Selfcare’, and ‘Interpersonal relationships’ were excluded from the analysis due to having only one variable remaining for the AMD group. Moreover, the scale reliability coefficients obtained were as follows: (1) For the item ‘Visibility’, the reliability coefficient was 0.6314. This value falls within the range considered acceptable for the internal consistency of the scale (0.6–0.7), suggesting that items related to visibility were moderately related and measured the same construct coherently. (2) For the item ‘Learning’, the reliability coefficient was 0.8642. This value is greater than 0.8, indicating high internal consistency among the items related to learning. This suggests that these items were highly related and measured the same construct consistently and reliably. (3) For the item ‘Mobility’, the reliability coefficient was 0.8511. Similar to the ‘Learning’ item, this value also exceeds 0.8, indicating high internal consistency among the items related to mobility. (4) For the item ‘Domestic Life’, the reliability coefficient was 0.7744. Although this value is above 0.7, it does not reach the threshold of 0.8 to be considered excellent. However, it remains acceptable and suggests moderate internal consistency among items related to domestic life. (5) Interestingly, the rural population (n = 109) considered the ‘Domestic Life’ item with two variables, resulting in an average interitem covariance of 0.2966 and a scale reliability coefficient of 0.7428. These values suggest moderate internal consistency, which is relatively acceptable.

For AMD people living in urban areas (n = 258), only the ‘Mobility’ item was considered, with an accepted average interitem covariance of 0.2753 and a scale reliability coefficient of 0.7243. Table 3 also indicates differences between older participants with AMD and older people not affected by AMD. For example, the analysis for persons without AMD in rural areas (n = 746) excluded the ‘Learning’ item. In contrast, urban areas excluded the items ‘Communication’ and ‘Learning’. However, ‘Communication’ was included in the rural and urban analyses.

## 4. Discussion

This study aimed to investigate how adults aged 50 and above—diagnosed with AMD and residing in rural and urban areas of Spain—perceived the impact of new technologies on the QoL items. The main findings focused on the response to the statement ‘Do you believe the use of new technologies has improved aspects of your daily life?’ 

Firstly, it has been well documented that aging is associated with an increase in diseases or issues related to mobility [45,46,47]. Our findings revealed a higher prevalence of AMD among women than men. While the distribution of ocular conditions such as glaucoma, cataracts, or diabetic retinopathy varies by sex, women with visual impairments constituted the majority. Approximately two out of every three persons in these categories were women with visual impairments [48]. This sex difference may be partly attributed to women’s longer life expectancy, which averages five years more than that of men [49]. Other factors, including disparities in the significance of optimal vision in daily activities, variations in the inclination to seek medical attention, and sex and/or gender disparities in access to healthcare services, could have also contributed to this disparity. Furthermore, lifestyle-related factors such as smoking habits and sun exposure may differ by sex, influencing the risk and distribution of ocular diseases among men and women. Moreover, sex-linked biological differences can influence the underlying pathogenic mechanisms of diseases [50]. Lastly, a technological divide was observed among older persons, and there were sex inequalities influenced by differences in biases. This disparity is particularly evident in women with disabilities. This study’s results revealed that how women perceive technologies has the potential to enhance their QoL and overall well-being, specifically in relation to AMD. Women see technological advancements as mitigating the impacts on autonomy and independence and improving their ability to manage their health. Additionally, women often engage in more caregiving activities, which can be physically and emotionally demanding. Technologies assisting in these areas provide significant relief and support [36]. For example, technologies such as Instead Technologies for Helping People (see EGARA handle) or rehabilitation neurorobotics for people that experienced stroke (see iDRhA) are specialized by grouping by sex [51].

Despite this positive outlook, prevailing social stereotypes and images present a conflicting narrative. These societal views often depict women, especially older women, as less capable of using or benefiting from new technologies [36]. This contradiction highlights the gap between women’s actual perceptions and experiences and the cultural, educational, and societal expectations. This also indicates the importance of rethinking about how new generations and intergenerational programs interact with older women, their demands, and the necessity for various technologies. This discrepancy influences how technologies are developed and scaled, potentially ignoring the real needs and insights offered by women about their health management and caregiving roles [52,53] in patients with AMD and family caregivers. For instance, social images and representations of older women tend to be characterized by stereotypes and discriminatory idealizations in relation to the understanding and use of technologies. As a result, older women are often rendered invisible in society and from its own images [52,53,54].

Another factor to take into consideration is the age-related sociocultural discrimination and antifeminist bias that women face in caregiving roles. Women continue to bear the greatest burden of care, with very few men participating in caregiving activities. These burden extent thought a woman’s life, including during old age and disability. Consequently, older women have a tendency to minimize the impact of their health and inequalities they face in comparison to older men. This phenomenon is evident in the responses provided on the Likert scale, where older women expressed that technology had no significant impact on either improving or worsening their daily lives. This limitation is also reflected in the current study, given that the number of male participants was smaller than that of women, potentially influencing the outcomes of the independent variables concerning sex differences. A second phase of this questionnaire that includes sex differences based on stratifying the analysis by age group would be helpful. This segmentation process can yield a more profound comprehension of how age impacts the associations between variables and uncover age-specific trends or patterns within the dataset. Moreover, expanding the male sample size will ensure a more equitable sex distribution, thereby proving advantageous. This would help mitigate the potential biases arising from the unequal representation of the sexes in the sample and enhance the generalizability of the findings. Furthermore, it is important to highlight that this research specifically concentrated on physiological sex distinctions and did not encompass a wider exploration of gender-related issues, encompassing social, cultural, and identity aspects.

On the other hand, our findings indicate that the majority of respondents, both in rural and urban areas, believed that new technologies’ use had not significantly altered their daily lives. However, a larger proportion of persons residing in urban areas claimed technology had improved certain aspects of their daily life compared to those in rural areas, which aligns with our expectations. AMD significantly impacts multiple aspects of daily functioning and the ability to live independently, encompassing essential activities such as reading, shopping, driving, and self-sufficient cooking [55]. The differences found between rural and urban areas were consistent with the results of a study conducted by Święch et al. 2021, which identified variations among subpopulations of patients with AMD residing in rural and urban environments [25]: persons with disabilities in rural areas frequently reported difficulties in reading. This suggests that the prescription of low-vision devices might be less frequent in rural areas, possibly due to a lack of detailed information provided to patients in these areas. Additionally, rural patients perceived that their vision loss significantly impacted their QoL more than persons living in urban areas. This difference may be attributed to urban areas being better adapted to persons with low vision, likely due to the widespread use of new technologies [25,56]. Rural areas present a multitude of additional challenges. Hence, our results indicate that in rural zones, a lower percentage of persons responded affirmatively to the question ‘Yes, technology has improved my daily life a lot’ and a higher percentage of persons who asserted that ‘Technology has not significantly changed my daily life’. This contrast is explained by the unequal access to advanced health services, which are more accessible in urban areas and may include telehealth and remote monitoring technologies, essential elements for the effective management of AMD. Additionally, internet connectivity, which facilitates access to information and online health services, tends to be more robust and reliable in urban areas. The complexity of rural challenges in medical aid are increased by insufficient internet connectivity and the persistent digital divide, which impact older people residing outside urban regions. These medical aids include mobility issue opportunities and risks. These risks encompass not only healthcare disparities for older people [57] but also the profound impacts of issues such as loneliness, isolation, and the various social challenges arising from the broader context of social distancing [58]. The sociocultural environment also influences the acceptance and adoption of new technologies and varies considerably between these areas, with those in rural areas often showing greater reluctance to adopt technological innovations due to cultural norms and resistance to change [57]. These multifaceted problems underscore the complex landscape faced by persons in rural settings, where a combination of factors, including limited healthcare access, technological disparities, and the broader sociocultural environment, collectively contribute to a distinct set of challenges that significantly influence the well-being of the population. Nevertheless, persons with low vision may experience increased insecurity in urban areas due to the increased mobility in urban settings.

More women expressed that new technologies improved their QoL than men. However, more women also believed that their daily life activities had neither improved nor worsened. This may also have been due to a limitation of this study, as there were more women than men included in the survey. For that reason, sex differences and a gendered approach may play a crucial role in how technology is perceived and used among older adults, underscoring the importance of considering gender differences in future research and in the development of technological interventions.

On the other hand, the findings of this study highlight significant differences in the perception of new technologies intended to enhance the daily QoL among older persons affected by AMD in urban and rural settings. Specifically, there was a notable positive impact observed in QoL aspects such as ‘Domestic life’, ‘Learning’, and ‘Mobility’, though with a less pronounced impact on the ‘Visibility’ item. In rural areas, a particularly positive perception was emphasized for ‘Domestic life’, reflecting the importance and demand for this item in such environments. Moreover, technology plays a crucial role in transforming domestic life. Although technological advancement has improved the living conditions for people in rural areas, there is still a significant gap in technology access and adoption, especially among older populations. This paper demonstrates that in rural older people affected by AMD, technology was positively impacting their learning abilities. This suggests that the digital divide may be narrowing in this population. On the other hand, according to the results obtained, there was no evidence that other factors such as mobility or communication impacted the QoL of people with AMD, consequently reducing the digital divide experienced by these persons.

Conversely, in urban areas, ‘Mobility’ emerged as the sole item considered in the analysis, underscoring the differences in the needs and priorities of persons according to their geographical context. In urban areas where there is the constant movement of people, mobility was a key factor intertwined with technology, which positively affected the QoL of older individuals with AMD. However, a different kind of divide was found, one centered around communication and learning, as observed in the results of this study.

For individuals aged 50 years and over in both rural and urban environments, the results showed that technological inclusion may have been favorably impacting learning and mobility, bridging the digital divide among older people with this condition. Addressing this challenge will require efforts by policymakers, public institutions, educators, and technology providers to emphasize solutions based on specific educational initiatives, accessible technology training programs [59,60], and inclusive design principles, aimed at empowering older people with technology. This suggests that future research on technology use and its impact on QoL in terms of mobility items should be related to the type of technological aid/device, which could be beneficial in uncovering the differences between areas and from a gender perspective.

Moreover, the data revealed that while older persons with AMD valued items related to ‘Learning’, those without this condition did not perceive a significant impact in this area. This finding suggests that AMD might specifically influence the perception and value placed on learning ability in adults and older adults, emphasizing the need for personalized and individually adapted strategies and training tailored to improve intrinsic capacity in promoting healthy aging. The necessity for personalized approaches becomes evident when considering these differences, both in the comparison between urban and rural settings and among persons with and without AMD. Therefore, previous studies analyzed technological opportunities for blind and visually impaired people [61]. This highlighted the specific impact of AMD on QoL and the importance of considering the individual and intrinsic capacity context when designing and implementing support technologies and programs, as stated by LaMonica et al., 2021 [62]. There are several strategies that can enhance the acceptance and effectiveness of technologies by the older adult community, including the use of codesign methodologies to ensure technology usability, acceptability, and efficiency. Telemedicine (e.g., virtual consultations, virtual rehabilitation programmes, remote diagnosis and patient monitoring and, emergency assistance) has emerged as an effective alternative for managing AMD, leveraging telecommunication technologies for remote treatment and patient assessment. This method includes synchronous and asynchronous care, communication between doctors and patients, and remote monitoring, allowing physicians to make accurate diagnoses and manage treatment from a distance [63] Additionally, the integration of mobile applications for visual self-monitoring and artificial intelligence for early detection has strengthened this strategy, enhancing diagnostic efficiency and access to medical care, reducing costs, and optimizing clinical time for cases requiring more intensive direct intervention [64].

During the COVID-19 pandemic, the emerging need to utilize technological services for socio-healthcare services, such as telemedicine, which has played a significant role, was clearly demonstrated, impacting patients with AMD. However, the current challenge extends beyond the immediate health crisis and calls for a holistic approach that exploits technology’s capabilities to enhance medical care and the QoL for patients, particularly those with chronic conditions like AMD. In this context, it is important to highlight that COVID-19 has underscored the significance in ensuring uninterrupted and safe medical care, reducing virus exposure, and improving healthcare accessibility [34].

The pandemic has indeed heightened concerns regarding the emergence of comorbidities. For individuals already afflicted with chronic ailments like AMD, the repercussions can be more severe as they encounter a heightened risk of complications and a potential decline in QoL [65,66,67].

The current challenge extends further, and it is necessary to continue promoting new technological advancements in healthcare, beyond telemedicine. This could encompass the development and implementation of artificial intelligence algorithms [51] based on machine learning and deep learning, aiding in the early detection, precise diagnosis, and personalized treatment of diseases like AMD. Moreover, disruptive technologies such as quantum computing open new possibilities for research and the development of more effective treatments.

Our study’s reliance on self-reported data potentially introduced biases, as participants may have inaccurately represented their technology usage and perceptions. The cross-sectional design additionally restricted our ability to establish causality between technology use and QoL improvements in older patients with AMD. Additionally, the representativeness of our sample may have affected the generalizability of our findings due to potential demographic and regional disparities. To address these issues, future research should include longitudinal studies to evaluate the long-term effects of technology interventions on QoL among older adults with AMD. Moreover, qualitative research is needed to uncover the reasons behind the sex differences in technology perception and usage, which will help tailor more effective interventions for this population in the clinic context. These interventions could include tailored technological solutions that are sensitive to the unique needs of this population, potentially influencing policy and practice in healthcare settings focused on aging populations [61,62]. To improve technology access in rural areas, strategies could include enhancing broadband infrastructure, providing incentives for technology companies, and increasing digital literacy through targeted education programs. Additionally, fostering partnerships between technology firms and healthcare providers and advocating for policies that promote technological equity are essential. These measures aim to bridge the gap in technology utilization, ensuring better health outcomes and QoL for rural communities.

Finally, our results underscore the importance of public policies and intervention strategies tailored to sex differences and geographic dispersion. Designing accessible technological solutions is crucial for promoting autonomy and enhancing the QoL among older adults, irrespective of their location. Moreover, gaining a comprehensive understanding of their perception is essential for developing supportive technologies that improve QoL. By considering these factors, technologies to facilitate interventions should be designed to be more aligned with the needs of older adults, promoting the management of their conditions and ultimately enhancing their overall well-being. In the future, addressing these gaps and harnessing technology’s capabilities will require coordinated efforts from policymakers, healthcare providers, educators, and technology developers. This collaboration should aim to ensure equal access to technology and personalized interventions for older adults with AMD. By prioritizing inclusivity and innovation, we can effectively use technology to improve the overall well-being and independence of older populations, promoting healthy aging for all.

## 5. Conclusions

This research demonstrated a correlation between technology perception and the improvement in the QoL of adults and older people. The distinction between urban and rural settings suggests substantial discrepancies in accessibility and technological impact. The greater appreciation for technology in persons affected by AMD and urban residents highlights the need for inclusive policies to enhance access to and utility of technologies in rural areas and for people with AMD. Although there was generally a positive perception of technology, the variations based on the presence of AMD and geographical location emphasize the importance of considering these differences in the development of technological interventions. Significant sex differences in technology perception were observed, with women reporting a more positive impact. This suggests that interventions should be sex-sensitive to maximize effectiveness.

This study revealed that those with AMD perceived technology as improving their condition, which was associated with various aspects of QoL, particularly affecting ‘Visibility’, ‘Learning’, ‘Mobility’, and ‘Domestic life’. Notably, rural populations assessed ‘Domestic life’ with two variables, while urban areas focused primarily on ‘Mobility’. These findings underscore the need for context-specific evaluations in QoL assessments, taking into account geographic location and AMD status. However, these perceptions are also influenced by the presence of comorbidities, which can exacerbate the problems associated with AMD.

Finally, this research provides a basis for future studies to explore the barriers and facilitators of technological adoption among older adults with AMD, with a particular focus on the intersection of sex, technology, and rural residence. Implementing these findings into practical applications and policy development would greatly aid in the inclusion of this demographic group into digital and social spheres, aligning with the Sustainable Development Goals focused on ensuring a dignified and high-quality life for all older people.

## Figures and Tables

**Table 1:** Sociodemographic variables in older people with or without AMD distributed in rural and urban areas.

Sociodemographics Variables			Totals (n = 2405)		Rural Areas (n = 855) ˂ 20,000 Habitants		Urban Areas (n = 1550) ≥ 20,000 Habitants	
		Totals (n = 2405)	AMD (n = 367)	Non-AMD (n = 2038)	AMD Persons (n = 109)	Non-AMD Persons (n = 746)	AMD Persons (n = 258)	Non-AMD Persons (n = 1292)
		[Mean ± SD]	[Mean ± SD]	[Mean ± SD]	[Mean ± SD]	[Mean ± SD]	[Mean ± SD]	[Mean ± SD]
		[N(%)]	[N(%)]	[N(%)]	[N(%)]	[N(%)]	[N(%)]	[N(%)]
**AMD diagnosis**	**Persons with AMD diagnosis**	367 (15.26)	367 (100)	--	109 (100)		258 (100)	--
	**Persons with non-AMD diagnosis**	2038 (84.74)	--	2038 (100)	--	746 (100)	--	1292 (100)
**Sex**	**Men**	870 (36.55)	110 (29.97)	760 (37.29)	33 (30.28)	292 (39.14)	77 (29.84)	468 (36.22)
	**Women**	1535 (63.83)	257 (70.03)	1278 (62.71)	76 (69.72)	454 (60.86)	181 (70.16)	824 (63.78)
**Age (≥50 years)**	**Total (n = 2405)**	79.20 ± 0.27	79.29 ± 0.63	73.27 ± 0.29	77.38 ± 1.18	74.77 ± 0.47	80.24 ± 0.74	72.40 ± 0.36
	**Women (n = 1535)**	72.81 ± 0.43	77.94 ± 1.14	72.08 ± 0.46	79.22 ± 1.33	75.32 ± 0.61	80.34 ± 0.92	73.23 ± 0.45
	**Men (n = 870)**	74.99 ± 0.34	80.01 ± 0.76	73.97 ± 0.37	73.12 ± 2.27	73.91 ± 0.73	80.01 ± 1.25	70.93 ± 0.59

AMD: age-related macular degeneration. --: Not applicable.

**Table 2:** Diagnoses related or unrelated to vision pathologies in rural and urban areas.

				Total (n = 2405)				Rural Areas (n = 855)				Urban Areas (n = 1550)			
		Total (n = 2405)		AMD (n = 367)		Non-AMD (n = 2038)		AMD Persons (n = 109)		Non-AMD Persons (n = 746)		AMD Persons (n = 258)		Non-AMD Persons (n = 1292)	
		N	% total	N	% AMD	N	% Non-AMD	N	% AMD	N	% Non-AMD	N	% AMD	N	% Non-AMD
**Diagnosis related to vision**	*Retinitis pigmentosa*	74	3.08	15	4.09	59	2.89	6	5.50	25	3.35	9	3.49	34	2.63
	*Magma myopia*	257	10.69	56	15.26	201	9.86	17	15.60	74	9.92	39	15.12	127	9.83
	*Diabetic retinopathy*	150	6.24	21	5.72	129	6.33	5	4.59	45	6.03	16	6.20	84	6.50
	*Glaucoma*	345	14.35	73	19.89	272	13.35	26	23.85	94	12.60	47	18.22	178	13.78
	*Cataract*	1207	50.19	195	53.13	1012	49.66	56	51.38	381	51.07	139	53.88	631	48.84
**Diagnosis non-related to vision**	*Cancer/malignant tumor*	268	11.14	50	13.62	218	10.70	16	14.68	76	10.19	34	13.18	142	10.99
	*Diabetes*	678	28.19	83	22.62	595	29.20	31	28.44	221	29.62	42	16.28	374	28.95
	*Chronic depression*	464	19.29	72	19.62	392	19.23	25	22.94	118	15.82	47	18.22	274	21.21
	*Chronic anxiety*	423	17.59	53	14.44	370	18.16	21	19.27	110	14.75	32	12.40	260	20.12
	*Parkinson*	80	3.33	13	3.54	67	3.29	5	4.59	16	2.14	8	3.10	51	3.95
	*Alzheimer*	260	10.81	27	7.36	233	11.43	10	9.17	46	6.17	17	6.59	87	6.73
	*Muscular dystrophy*	149	6.20	25	6.81	124	6.08	10	9.17	37	4.96	15	5.81	87	6.73
	*Stroke*	250	10.40	36	9.81	214	10.50	13	11.93	86	11.53	23	8.91	128	9.91
	*Myocardial infarction*	222	9.23	38	10.35	184	9.03	12	11.01	72	9.65	26	10.08	112	8.67
	*Arthritis*	720	29.94	124	33.79	596	29.24	35	32.11	228	30.56	89	34.50	368	28.48
	*Osteoarthritis*	1242	51.64	221	60.22	1021	50.10	61	55.96	382	51.21	160	62.02	369	28.56

AMD: Age-related macular degeneration.

**Table 3:** Average interitem covariance, number of items, and scale reliability coefficients.

	Total (n = 2405)						Rural Areas (n = 855) ˂ 20,000 Habitants						Urban Areas (n = 1550) ≥ 20,000 Habitants					
	AMD (n = 367)			Non-AMD (n = 2038)			AMD Persons (n = 109)			Non-AMD Persons (n = 746)			AMD Persons (n = 258)			Non-AMD Persons (n = 1292)		
ITEM	Average Interitem Covariance	Number of Items in the Scale	Scale Reliability Coefficient	Average Interitem Covariance	Number of Items in the Scale	Scale Reliability Coefficient	Average Interitem Covariance	Number of Items in the Scale	Scale Reliability Coefficient	Average Interitem Covariance	Number of Items in the Scale	Scale Reliability Coefficient	Average Interitem Covariance	Number of Items in the Scale	Scale Reliability Coefficient	Average Interitem Covariance	Number of Items in the Scale	Scale Reliability Coefficient
**Visibility**	0.1938 ^1^	3	0.6314 ^2^	0.2628 ^1^	3	0.7353 ^2^	---	1	---	0.0742	6	0.2628	---	1	---	0.2560 ^1^	3	0.7181 ^2^
**Communication**	---	1	---	0.3244 ^1^	6	0.8741 ^3^	---	0	---	0.3816 ^1^	5	0.8886 ^3^	---	0	---	---	0	---
**Learning**	0.3410 ^1^	7	0.8642 ^3^	0.2225 ^1^	4	0.7573 ^2^	---	0	---	---	1	---	---	0	---	---	0	---
**Mobility**	0.2799 ^1^	8	0.8511 ^3^	0.2781 ^1^	14	0.9119 ^3^	---	1	---	0.2494 ^1^	10	0.8812 ^3^	0.2753 ^1^	4	0.7243 ^2^	0.2881 ^1^	9	0.8772 ^3^
**Selfcare**	---	1	---	0.3573 ^1^	9	0.8806 ^3^	---	0	---	0.5014	6	0.8535 ^3^	---	1	---	0.2651 ^1^	3	0.7213 ^2^
**Domestic life**	0.3815 ^1^	3	0.7744 ^2^	0.3983 ^1^	6	0.8474 ^3^	0.2966 ^1^	2	0.7428 ^2^	0.3848 ^1^	5	0.8314 ^3^	---	1	---	0.4026 ^1^	6	0.8490 ^3^
**Interpersonal relationships**	---	0	---	0.2399 ^1^	4	0.7646 ^2^	---	0	---	0.3043 ^1^	3	0.7937 ^2^	---	0	---	0.2882 ^1^	2	0.8094 ^3^

Note: ^1^ Average interitem covariance: 0.15 to 0.5; scale reliability coefficient: ^2^ acceptable = 0.6–0.8. ^3^ Value > 0.8. AMD: Age-related macular degeneration. ---: excluded from the analysis.

## Data Availability

The data from the “Survey of Disability, Personal Autonomy and Dependency Situations (2020)”, developed by the National Institute of Statistics (INE), are available at https://www.ine.es/dyngs/INEbase/en/operacion.htm?c=Estadistica_C&cid=1254736176782&idp=1254735573175, accessed on 2 February 2024. The methodology employed for the questionnaire encompassed various aspects related to QoL based on the WHO dimensions of the WHOQOL-100 scale, available at https://www.who.int/tools/whoqol/whoqol-100, accessed on 5 February 2024.

## Supplementary Materials

The following supporting information can be downloaded at: https://www.mdpi.com/article/10.3390/ejihpe14050097/s1, Table S1: Items: ‘Difficulty level, difficulty level for/to’.
