# Supplementary material for: Technological Perception with Rural and Urban Differentiation and Its Influence on the Quality of Life of Older People with Age-Related Macular Degeneration
Source: Eur J Investig Health Psychol Educ. 2024 May 20;14(5):1470–88. doi: 10.3390/ejihpe14050097 (PMC11119705; doi:10.3390/ejihpe14050097)
Supplement: Supplementary file 1 [file ejihpe-14-00097-s001.zip › ejihpe-2924143-supplementary.pdf]

Table S1. 'Items: Difficulty level, difficulty level for/to'...

		Difficulty level, difficulty level for/to...
VISIBILITY	visi_2_2	... see newspaper print
	visi_2_4	... see newspaper print when you use technical aids
	visi_4_2	... you have with that or another vision problem
	visi_4_4	... you have when you use technical aids
COMUNICATION	comu_8_2	...speak in an understandable way or say sentences that make sense
	comu_9_2	...understand the meaning of what others say
	comu_9_4	...understand the meaning of what others say to you when you receive help
	comu_10_2	...understand and express themselves through written language
	comu_11_2	...understand and express themselves through gestures, symbols, drawings or sounds
	comu_12_2	...hold a dialogue and exchange ideas with one or more people
	comu_14_2	...use remote written communication systems
LEARNING	apre_15_2	...pay attention with your eyes or keep your attention with your ears
	apre_16_2	...learn to do simple things
	apre_17_2	...carry out simple tasks
	apre_18_2	...carry out complex tasks
MOBILITY	movi_19_2	...change position
	movi_20_2	...keep the body in the same position
	movi_21_2	...walk or move around your home
	movi_21_4	...walk or move around in your home when you receive help or personal assistance
	movi_22_2	...walk or move outside your home
	movi_22_4	...walk or move outside your home when you receive help or personal assistance
	movi_23_2	...move using means of transport as a passenger
	movi_23_4	...move using means of transport as a passenger when receiving personal help or assistance
	movi_24_2	...driving vehicles without adaptations
	movi_25_2	...manipulate and move objects, using hands and arms
	movi_25_4	...manipulate and move objects, using hands and arms when receiving personal help or assistance
	movi_26_2	...manipulate small objects with hands and fingers
	movi_26_4	...manipulate small objects with hands and fingers when receiving personal help or assistance
SELFCARE	auto_27_2	...wash and dry the different parts of the body
	auto_27_4	...wash and dry the different parts of the body when receiving help or personal assistance
	auto_28_2	...perform basic body care
	auto_28_4	...perform basic body care when receiving personal help or assistance
	auto_29_2	...go to the toilet and relieve yourself or take care of your intimate hygiene
	auto_30_2	...dress or undress
	auto_31_2	...eat and drink
	auto_32_2	...comply with medical prescriptions
	auto_32_4	...fulfill medical prescriptions when receiving personal help or assistance
	auto_33_2	...avoid dangerous situations
DOMESTIC LIFE	vdom_34_2	...manage the household budget, plan expenses or organize purchases
	vdom_34_4	...manage the household budget, plan expenses or organize purchases when you receive personal help or assistance
	vdom_35_2	...prepare meals
	vdom_35_4	...prepare meals when receiving personal help or assistance
	vdom_36_2	...take care of other chores around the house
	vdom_36_4	...take care of other chores around the house when you receive personal help or assistance
INTERPERSONAL	rela_37_2	...show affection, respect or feelings
	rela_38_2	...associating with unknown people
	rela_39_2	...create and maintain relationships with friends, neighbors, acquaintances, subordinates, superiors or colleagues
	rela_40_2	...form a family or maintain family relationships
